# Locoregional Lymph Node Metastasis from Clinically Occult Breast Cancer: Prognostic Significance of Mastectomy

**DOI:** 10.1155/2024/5878308

**Published:** 2024-08-09

**Authors:** Andreas Werner Nærum, Emil Villiam Holm-Rasmussen, Ilse Vejborg, Ann Søegaard Knoop, Anne-Vibeke Lænkholm, Niels Kroman, Tove Filtenborg Tvedskov

**Affiliations:** ^1^ Department of Breast Surgery Herlev-Gentofte Hospital, Copenhagen, Denmark; ^2^ Department of Plastic Surgery and Burns Treatment Rigshospitalet, Copenhagen, Denmark; ^3^ Department of Breast Examinations Herlev-Gentofte Hospital, Copenhagen, Denmark; ^4^ Department of Oncology Rigshospitalet, Copenhagen, Denmark; ^5^ Department of Surgical Pathology Zealand University Hospital, Roskilde, Denmark

## Abstract

**Materials and Methods:**

This study included patients registered in the national Danish Breast Cancer Group (DBCG) database between 2001 and 2015, with locoregional LNM as well as a bilateral negative mammography, ultrasonography, and physical examination of the breasts. Overall survival (OS) and invasive disease-free survival (IDFS) were compared by treatment groups, ALND + RT (axillary lymph node dissection and radiotherapy) or ALND + MAST ± RT (axillary lymph node dissection, mastectomy with or without radiotherapy).

**Results:**

In total, 56 patients were included in the study, of which 37 were treated by ALND + RT, 16 by ALND + MAST ± RT, and the remaining three patients receiving different treatments. The median follow-up for the 53 OBC patients sorted by treatment group was 12.2 years (interquartile range: 10.1 years; 15.3 years). There was no significant difference in OS or IDFS between the treatment groups, except for a subgroup of 46 (out of 53) patients without verified *in situ* lesions before treatment, where ALND + RT treatment showed an improved OS (log-rank *p*=0.05).

**Conclusion:**

Treating OBC patients with ALND and radiotherapy resulted in a similar outcome as treatment with ALND and mastectomy. This supports omission of mastectomy in favor of radiotherapy of the breast in these patients.

## 1. Introduction

Occult breast cancer (OBC) describes breast cancer metastases identified in locoregional lymph nodes without a corresponding identifiable malignant tumor in the breast. It was first described in 1907 as “cancerous axillary glands with nondemonstrable cancer of the mamma” [1]. The definition has since been expanded to include undetectable breast lesions by ultrasonography (US) and mammography (MG). The incidence of OBC accounts for less than one percent of all breast cancer cases [2, 3].

Magnetic resonance imaging (MRI) can identify a primary breast lesion otherwise deemed OBC in 36–86% of cases [4]. As technological advances are made in diagnostics, the incidence of this rare subgroup of cancer will likely decrease.

Evidence of incidence, prognosis, and treatment of OBC is limited, and no prospective randomized trials currently exist. Only minor cohort studies or larger register studies based on information from the Surveillance, Epidemiology, and End Results or the National Cancer Database have been published and no information on recurrence was collected [5–7]. Previous studies have shown that treatment of OBC with mastectomy or radiotherapy (RT) of the breast reduces local recurrence and improves survival compared to observation [8–10].

Nevertheless, the best treatment of the breast in OBC is still debated. When comparing treatment strategies, some studies found no difference in survival between OBC treated with mastectomy versus RT while others found a higher overall survival (OS) for OBC patients treated with axillary lymph node dissection (ALND) and RT compared to ALND and mastectomy [2, 5, 11]. However, these studies comparing locoregional strategies of OBC lack information on recurrence.

Based on the limited evidence available, Danish guidelines, described by the Danish Breast Cancer Group (DBCG), currently recommend treatment of OBC with ALND and RT of the breast without surgery [12]. DBCG has since 1977 systematically collected data on diagnosis, treatment, as well as recurrence and survival. Today the database contains information on more than 100,000 breast cancer patients and has a high degree of completeness [13–15]. The DBCG database offers a unique opportunity to collect more evidence on the most optimal treatment of OBC.

This study aims to determine the clinicopathological characteristics of clinical occult breast cancer (cOBC) presenting with locoregional lymph node metastasis (LNM) and to compare prognosis after different treatment options based on data from the DBCG database.

## 2. Materials and Methods

### 2.1. Study Design

This study is a nationwide retrospective study including all Danish women with breast cancer registered in the DBCG database between 2001 and 2015 with invasive breast cancer and tumor size 0 mm (“0”), 1 mm (“1”), “unknown” or “missing,” treated with primary surgery. Since 2000, Danish breast cancer patients have mainly been diagnosed in centralized settings with triple diagnostic tests, which are: breast examination, mammography, and ultrasound examination of the whole ipsilateral and contralateral breast, including the axilla with needle biopsy of suspicious areas. MRI can be used as a supplementary diagnostic tool when a primary tumor is undetectable, i.e., occult, following triple diagnostic tests [16].

### 2.2. Eligibility Criteria

Data retrieved from the DBCG database was validated through pathology reports from the national Pathology Data Bank as well as original patient files to verify that no invasive tumor was identified during the diagnostic process. In this study, we defined cOBC as patients with locoregional LNM from breast cancer and bilateral negative MG, US, and physical examination of the breasts. Patients fulfilling these criteria were included in the study. Patients with positive findings on MRI followed by a negative biopsy were also included, whereas patients with biopsy-verified malignancies or malignantly suspicious lesions without biopsy identified by MRI were excluded. Patients with a primary tumor identified in the mastectomy specimen were considered as having clinical occult breast cancer and were included as well. Excluding patients with breast cancer on final pathology of the mastectomy specimen would result in selection bias in the mastectomy group because a similar group could not be identified and excluded from the group of patients with RT only.

Patients with primary breast cancer detected by radiological or physical examination, distant metastatic disease at time of diagnosis, mastitis carcinomatosa, pathologically undetermined malignant breast lesions (either primary or metastatic), previous history of breast cancer (ipsilateral and/or contralateral), and/or ductal carcinoma *in situ* (DCIS) treated with RT of the breast and/or axilla, were all excluded from the study. The cohort of cOBC thus consists of TNM-stages T0N1-3M0 and TxN1-3M0.

### 2.3. Data Collection

Data on the following variables were collected: age at diagnosis, treatment (ALND + RT or ALND + MAST ± RT), estrogen receptor (ER) status, human epidermal growth factor receptor-2 (HER-2) status, number of resected lymph nodes, number of positive lymph nodes, use of US, MG, and MRI, use of RT (to the breast and axilla), and use of adjuvant systemic therapy (chemotherapy, endocrine treatment, or trastuzumab). Information on patient outcomes, including follow-up on recurrence of ipsilateral and/or contralateral breast cancer, regional or distant metastasis, other primary cancers, and date of death were collected from original patient files until the 28^th^ of May 2021. Patients with ≥10 lymph nodes resected were considered treated with ALND. ER status was defined as positive if ≥10% of the tumor cells stained positive by immunohistochemistry (IHC) and negative if <10%. HER-2 status was defined according to national guidelines [17]. Strong HER-2 IHC expression (3+) were defined as positive, while 0, and 1+ were defined as negative. A borderline HER-2 expression status of 2+ was further analyzed by *in situ* hybridization, and HER-2 amplification was determined by a ratio of ≥2.0. Since primary lesions were unavailable, ER and HER-2 analyses were performed on the LNM.

### 2.4. Data Analysis and Endpoints

Patients were categorized into subgroups based on treatment strategy (ALND + RT and ALND + MAST ± RT). The primary endpoint was overall survival (OS), defined as time between diagnosis and death from all causes. Invasive disease-free survival (IDFS) was measured as a secondary endpoint, as well as evaluation of the clinicopathological characteristics of cOBC. IDFS was defined according to the STEEP system [18]. OS and IDFS were also measured on a subgroup of cOBC, excluding patients found to have DCIS during the diagnostic process, as well as patients with a history of DCIS.

### 2.5. Statistical Analysis

Descriptive statistics were used for demographics and clinicopathological characteristics of cOBC. Fisher's exact test was used to examine differences in categorical variables in the treatment groups. Survival curves of the treatment groups were calculated using the Kaplan–Meier estimator and compared using the log-rank test to examine differences in OS and IDFS. Median follow-up was calculated using the reverse Kaplan–Meier estimator. The effects of breast treatment on OS and IDFS were quantified in terms of unadjusted, MRI-adjusted, and age-adjusted hazard ratios (HRs) using the Cox proportional hazards model. The same tests were used on the subgroup of cOBC described in “Data analysis and endpoints.”

A *p* value of ≤0.05 was considered statistically significant. Statistical analysis was performed using the statistical software RStudio (version 1.4.1717).

### 2.6. Approval

The study is registered at Videnscenter for Dataanmeldelser (P-2021-24) and approved by the Centre for Regional Development in the Capital Region of Denmark (R-20083112). Approval to use the DBCG data was granted by The Danish Clinical Quality Program–National Clinical Registries (RKKP). The data were originally linked to the case number DBCG-2016-01, but permission to use the data for this study was granted by RKKP.

## 3. Results

### 3.1. Patient Characteristics

**Figure 1:**
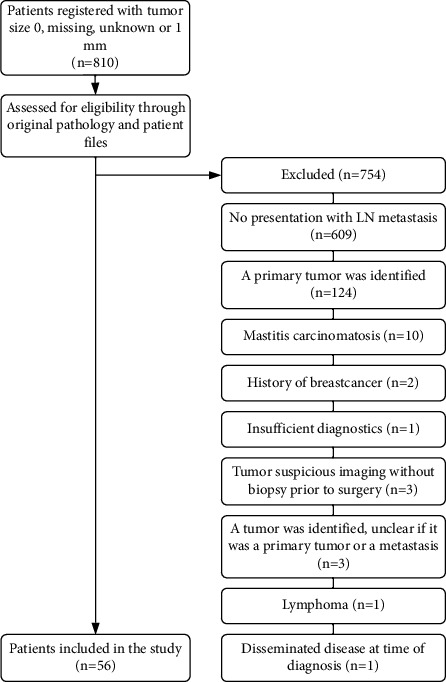
Flowchart for inclusion of occult breast cancer patients from the DBCG database 2001–2015. LN, lymph node(s).

From 2001 to 2015, 810 patients were registered in the DBCG database with tumor size “0,” “1,” “unknown” or “missing.” Fifty-six of these patients met the eligibility criteria and were included in the study (Figure 1). Median age at time of diagnosis was 63 years (range: 34–87 years), and 44.6% of patients were ER negative, and 21.4% were HER-2 positive. While thirty-one patients were ER positive, thirty-five received endocrine treatment, due to some centers using >1% stained tumor cells as indication for endocrine treatment. Approximately half of the patients received an MRI. Among patients treated with mastectomy (*n* = 16), four (25.0%) had an invasive carcinoma, and four (25.0%) had carcinoma *in situ* identified during pathological examination. It should be noted that all four patients with invasive carcinoma identified on postoperative pathology did not receive a preoperative MRI. Disease and treatment characteristics are shown in Table 1.

### 3.2. Treatment Groups

In total, 37 patients were treated with ALND + RT, 16 patients were treated with ALND + MAST ± RT, and two patients underwent lumpectomy, as they presented with intramammary LNM. One patient previously underwent mastectomy of the same breast due to a previous *in situ* lesion. Two of the 16 patients in the mastectomy group did not receive any RT to either the breast or axilla.

Patients in the ALND + RT group were more likely to have received an MRI (*p*=0.01) and significantly more patients were HER-2 positive in the ALND + MAST ± RT group (*p*=0.05). Patients diagnosed in 2010–2015 compared to 2001–2009 were more likely to have received an MRI (*p*=0.05) There was no significant association between treatment choice and year of diagnosis (2001–2009 compared to 2010–2015) (*p*=0.24).

### 3.3. Overall Survival

**Figure 2:**
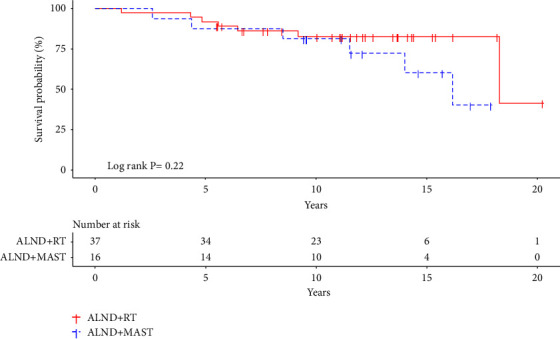
Kaplan–Meier plot displaying overall survival for 53 clinical occult breast cancer patients treated with ALND + RT (red line) and ALND + MAST ± RT (blue line). ALND, axillary lymph node dissection; RT, radiotherapy; MAST, mastectomy.

Patients treated with mastectomy or breast RT (*n* *=* 53) were included in the survival analysis. 13 of those died from all causes. The median follow-up was 12.2 years. Patients treated with ALND + RT had a 10-year OS rate of 82.6% (CI 95%: 70.8–96.5) and patients treated with ALND + MAST ± RT had a 10-year OS rate of 81.3% (CI 95%: 64.2–100.0; (log-rank *p*=0.22; Figure 2). In univariate analysis, no significant difference in OS was found (HR: 2.0; (CI 95%: 0.7–6.3; *p*=0.22). When adjusting for age (<50 or ≥50 at time of diagnosis) and the use of MRI, no significant difference in OS between groups was found (HR: 1.7; (CI 95%: 0.5–5.5; *p*=0.42 (Table 2). Neither age nor the use of MRI was independently associated with OS.

### 3.4. Invasive Disease-Free Survival

52 patients were included in the survival analysis of IDFS. One patient was excluded because of a primary nonbreast cancer diagnosis prior to the cOBC diagnosis. 19 patients had an event during follow-up. The median follow-up was 12.2 years. The 10-year IDFS rate for ALND + RT and ALND + MAST ± RT was 75.0% (CI 95%: 62.0–90.7) and 66.7% (CI 95%: 46.6–95.3; log-rank *p*=0.53), respectively. Univariate analysis showed no significant difference in IDFS between groups (HR: 1.4 CI 95%: 0.5–3.5; *p*=0.52). Likewise, no significant difference was found when adjusting for age or MRI use (HR: 1.2; CI 95% (0.4–3.2; *p*=0.74) (Table 2). Age or MRI use was not independently associated with IDFS.

### 3.5. Overall Survival and Invasive Disease-Free Survival excluding *In Situ* Lesions

When analyzing OS rate in a subgroup of OBC excluding *in situ* lesions (*n* = 46), patients treated with ALND + RT had a 10-year OS of 84.4% (CI 95%: 72.7–98.1), whereas ALND + MAST ± RT had a 10-year OS of 72.7% (CI 95%: 50.6–100.0). The median follow-up was 12.2 years. The OS rate of ALND + RT was significantly higher compared to ALND + MAST ± RT (log-rank *p*=0.05). However, univariate analysis showed no significant difference in the OS between groups (HR: 3.3; CI 95%: 0.95–11.4; *p*=0.06). Neither did survival analysis, when adjusting for age and MRI use (HR: 2.4; CI 95%: 0.6–8.9; *p*=0.20; Table 2).

Using the same limited cOBC criteria, treatment with ALND + RT had a 10-year IDFS rate of 76.4% (CI 95%: 63.2–92.3), whereas ALND + MAST ± RT had a 10-year IDFS rate of 63.6% (CI 95%: 40.7–99.5). This difference was not significant (log-rank *p*=0.29). The median follow-up was 12.2 years. Univariate analysis showed no significant difference in IDFS between treatment groups (HR: 1.7; CI 95%: 0.6–4.7; *p*=0.29), even if adjusted for age and MRI use (HR: 1.2; CI 95%: 0.4–3.4; *p*=0.77; Table 2).

## 4. Discussion

This study investigated the clinicopathological characteristics and outcomes of patients with cOBC treated with either RT or mastectomy. cOBC is a rare condition. According to data from The Danish Cancer Registry, 68,371 female breast cancer patients were diagnosed in Denmark between 2001 and 2015, resulting in an estimated cOBC incidence of only 0.08% based on the eligibility criteria in this study [19].

We found no significant differences in OS or IDFS when treating patients with mastectomy or breast RT, besides in the subgroup of patients without *in situ* lesions, where RT resulted in a higher OS rate. However, this was the only statistically significant finding in the survival analysis, as neither univariate nor multiple Cox regressions investigating OS in the same group were statistically significant. Accordingly, more research on this subgroup is needed.

Smaller studies with 29–53 patients and a short follow-up compared RT of the breast with observation in cOBC patients have found a reduced rate of local recurrence when treating with RT compared to observation [8–10]. Other studies found a significantly higher OS, disease-free survival and cause-specific survival when treating with RT [2, 5, 11, 20]. In contrast, one study found a similar OS in cOBC with or without surgery of the breast, but this study was compromised by a heterogenous cohort, where at least half of the group only treated with ALND also received RT [21].

Studies comparing RT of the breast to mastectomy in cOBC found no significant difference between treatment groups in 10-year OS despite one study reporting a higher 8-years OS when treating with RT instead of surgery [2, 5, 11]. Additionally, a meta-analysis including 241 patients from seven studies found similar OS, locoregional recurrence, and distant metastasis when comparing RT to mastectomy in cOBC [22]. Notably, some of these studies lack information on recurrence, HER-2 status, and neoadjuvant therapy [2, 5, 11]. The present study includes information on these variables and confirms a similar 10-year OS when treating cOBC patients with mastectomy or RT. This supports the DBCGs recommended treatment with RT of the breast without breast surgery for cOBC [2, 5, 11, 22].

The above-mentioned studies are limited by short follow-ups of 2–4 years [2, 5, 11, 22]. A significant strength of our study is the long follow-up of more than 12 years. This is crucial when studying cOBC recurrence, as small undetected breast lesions can present several years after initial treatment.

A meta-analysis of non-OBC breast cancer with more than 1,500,000 patients found that patients who underwent BCS had better OS compared to treatment with a mastectomy [23]. Keeping this as well as the results of this study in mind, clinicians discussing treatment options with OBC patients, should apply a “do no harm” approach and opt for the less invasive treatment of RT and avoid a mastectomy.

In this study, we chose to focus on cOBC, as this is the presentation of disease that clinicians need to treat, as pathological occult breast cancer (pOBC) with no detectable malignancy during pathological examination of the mastectomy specimen can only be assessed if the choice of treatment includes a mastectomy. The definition of cOBC is heterogenous and variably includes a negative MG, US, or MRI. Including a negative MRI in the definition of OBC in future studies is meaningful as MRI has been shown to detect primary tumors in otherwise occult cases in 72% of cases, with a sensitivity of up to 96% but a specificity of 63% [4, 24]. Specificity is reliant on several factors, including reader expertise [25]. Because of this, MRI-suspect breast lesions should be histologically confirmed using MRI-guided breast biopsy before deeming it non-OBC.

Our study is limited by the small sample size due to the low cOBC incidence. We used a nationwide dataset from the DBCG database, which has a completeness of more than 95% [15]. Despite the prospective and detailed registration in the database, many patients were excluded after register-based identification when validating data. This indicates that registration bias in OBC research is important and finding true OBC in large register-based studies can be challenging and generate invalid results. A strength of this study is the thorough validation and selection of patients.

This study has additional limitations. Patients were registered with OBC in the DBCG database *after* pathological examination. Thus, patients with a tumor identified at final pathology following mastectomy, could be identified as non-cOBC in the dataset. Studies have observed tumors identified postmastectomy in 20–74% of cases, depending on the slice interval and whether or not they had an MRI prior to surgery [20, 26–28]. If larger occult tumors are potentially missing from the mastectomy group, this selection bias would improve outcomes in the mastectomy group. However, of the sixteen patients treated with mastectomy, four had a DCIS and four had invasive carcinoma detected during pathological examination and were still present in our dataset. The aforementioned selection bias can also alter the estimated cOBC incidence. Therefore, the accurate cOBC incidence among Danish women is likely higher than 0.08%. Only eight of sixteen patients in our study treated with mastectomy had pOBC. Of these eight patients, seven did not receive a preoperative MRI. It is possible that a preoperative MRI could have assisted the pathologist in identifying small malignant lesions in the breast in these patients.

Patients in our study had significantly higher probabilities of being treated with RT if they had an MRI prior to treatment. This means that the mastectomy group could have unnoticed MRI-detectable breast lesions, potentially influencing the survival rate of the mastectomy group. However, it is unclear if this would significantly alter the outcome of the study, as tumors detected during pathological examination were unlikely to be included in our data. The association between MRI and RT could partially be explained by MRI availability in relation to year of diagnosis, as MRI was introduced in Danish breast cancer imaging from 2003 to 2011 (Personal communication). There was a trend toward RT rather than mastectomy in the later years of the study period, but this was not statistically significant.

We found an unexplained higher proportion of HER-2-positive patients in the mastectomy group. HER-2 positivity is associated with a worse prognosis and this might have influenced the choice of treatment [29]. Despite this, there was no significant association of trastuzumab treatment between treatment groups.

Because of the unidentifiable primary tumor in cOBC, biological characteristics of the cancer and choice of treatment will often be determined based on the receptor status of the locoregional LNM. Previous studies have investigated the rate of receptor discordance between axillary LNM and the primary tumors in breast cancer, with ER and HER-2 having a discordance rate of 0–28.8% and 0–13.5%, respectively [30–35]. Discordance rates could challenge the optimal cOBC treatment.

Keeping receptor discordance in mind, cOBC have been shown more often to be ER-negative and HER-2-positive, compared to non-cOBC [36]. Our study found a high ER-negative rate of 44.6% among cOBC, compared to only a 15–20% ER negative rate amongst non-cOBC [17]. We found a HER-2 positive rate of 21.4% amongst cOBC compared to the 10–15% HER-2 overexpression amongst non-cOBC described in the DBCG data [17]. However, a large portion of our patients had missing HER-2 status due to the introduction of HER-2 assessment as standard in Denmark in 2010 [14]. The increased rate of ER-negative and HER-2 positive tumors among cOBC suggests a more aggressive tumor profile compared to non-cOBC. This could explain small, nondetectable breast lesions presents with LNM. Further research, including a non-cOBC control group, would be beneficial to achieve knowledge of the cOBC tumor profile.

## 5. Conclusion

In conclusion, we found that patients presenting with locoregional LNM from an cOBC had similar prognosis in terms of overall survival and recurrence when treated with ALND and RT of the ipsilateral breast compared to ALND, RT, and mastectomy. The results support the DBCG guidelines and add to the validity of treating cOBC with RT of the breast without mastectomy. In the future, mastectomy should be omitted in cOBC in favor of breast RT, achieving a better cosmetic result without compromising the survival of the patient.

## Figures and Tables

**Table 1:** Patient, disease, and treatment characteristics of Danish cOBC patients diagnosed between 2001 and 2015 according to treatment of the breast.

	Total (*N* = 56)		ALND + RT (*N* = 37)^a^		ALND + MAST (*N* = 16)^a^		*p* value^b^
	*N*	%	*N*	%	*N*	%	
Age at diagnosis							
<50	11	19.6	7	18.9	3	18.8	1.00
≥50	45	80.4	30	81.1	13	81.2	
MRI							
Yes	31	55.4	26	70.3	4	25.0	**0.01**
No	25	44.6	11	29.7	12	75.0	
Receptor status							
ER positive	31	55.4	20	54.1	10	62.5	0.76
ER negative	25	44.6	17	45.9	6	37.5	
HER-2 positive	12	21.4	6	16.2	5	31.2	**0.05**
HER-2 negative	32	57.1	25	67.6	5	31.2	
HER-2 unknown	12	21.4	6	16.2	6	37.5	
Nodal status							
1–3 positive nodes	32	57.1	19	51.4	11	68.8	0.37
≥4 positive nodes	24	42.9	18	48.6	5	31.2	
Endocrine treatment							
Yes	35	62.5	23	62.2	11	68.8	0.76
No	21	37.5	14	37.8	5	31.2	
Trastuzumab treatment							
Yes	8	14.3	5	13.5	3	18.8	0.69
No	48	85.7	32	86.5	13	81.2	
Chemotherapy							
Yes	33	58.9	22	59.5	10	62.5	1.00
No	23	41.1	15	40.5	6	37.5	
Radiotherapy							
Yes	53	94.6	37	100	14	87.5	0.09
No	3	5.4	0	0	2	12.5	

cOBC, clinical occult breast cancer; ALND, axillary lymph node dissection; RT, radiotherapy; MAST, mastectomy; MRI, magnetic resonance imaging; ER, estrogen receptor; HER-2, human epidermal growth factor receptor 2. *Note*. ^a^3 patients were not placed in the treatment groups because they received different treatments. ^b^The *p* value were calculated using Fisher's exact test. Values are displayed in bold in Table 1 when they are statistically significant.

**Table 2:** Multivariable Cox proportional hazards regression for overall survival and invasive disease-free survival in cOBC patients diagnosed between 2001 and 2015.

	OS		IDFS	
	HR (95% CI)	*p* value	HR (95% CI)	*p* value
OBC patients (*n* = 53)^a^				
Treatment with ALND + RT	Reference		Reference	
Treatment with ALND + MAST	1.7 (0.5–5.5)	0.42	1.2 (0.4–3.2)	0.74
MRI: no	Reference		Reference	
MRI: yes	0.6 (0.2–2.1)	0.38	0.6 (0.2–1.6)	0.28
Age ≥50 at time of diagnosis	Reference		Reference	
Age <50 at time of diagnosis	2.6 (0.3–20.2)	0.36	5.5 (0.7–41.7)	0.10
OBC patients, excluding *in situ* lesions (*n* = 46)^b^				
Treatment with ALND + RT	Reference		Reference	
Treatment with ALND + MAST	2.4 (0.6–8.8)	0.20	1.2 (0.4–3.4)	0.77
MRI: no	Reference		Reference	
MRI: yes	0.3 (0.1–1.4)	0.13	0.5 (0.2–1.3)	0.13
Age ≥50 at time of diagnosis	Reference		Reference	
Age <50 at time of diagnosis	1.1 (0.1–9.1)	0.96	3.1 (0.4–23.7)	0.29

cOBC, clinical occult breast cancer; OS, overall survival; IDFS, invasive disease-free survival; HR, hazard ratio; CI, confidence interval; MRI, magnetic resonance imaging; ALND, axillary lymph node dissection; RT, radiotherapy; MAST, mastectomy. *Note*. ^a^One patient was excluded from the analysis of IDFS, resulting in a population of 52 patients. ^b^46 patients were included in this analysis of both OS and IDFS.

## Data Availability

The datasets generated during and/or analysed in this study are made available from the corresponding author upon reasonable request.
